# OCT Intensity of the Region between Outer Retina Band 2 and Band 3 as a Biomarker for Retinal Degeneration and Therapy

**DOI:** 10.3390/bioengineering11050449

**Published:** 2024-05-01

**Authors:** Yong Zeng, Shasha Gao, Yichao Li, Dario Marangoni, Tharindu De Silva, Wai T. Wong, Emily Y. Chew, Xun Sun, Tiansen Li, Paul A. Sieving, Haohua Qian

**Affiliations:** 1 Visual Function Core, National Eye Institute, National Institutes of Health, Bethesda, MD 20892, USA; yong.zeng@nih.gov (Y.Z.); sunnyangel2009@hotmail.com (S.G.); yichao.li@nih.gov (Y.L.); 2 Department of Ophthalmology, The First Affiliated Hospital of Zhengzhou University, Zhengzhou 450052, China; 3 Section for Translational Research in Retinal and Macular Degeneration, National Institute on Deafness and Other Communication Disorders, National Institutes of Health, Bethesda, MD 20892, USA; dario.marangoni@units.it; 4 Unit on Clinical Investigation of Retinal Disease, National Eye Institute, National Institutes of Health, Bethesda, MD 20892, USA; 5 Section on Neuron-Glia Interactions in Retinal Disease, National Eye Institute, National Institutes of Health, Bethesda, MD 20892, USA; wongw@alum.mit.edu; 6 Clinical Trials Branch, National Eye Institute, National Institutes of Health, Bethesda, MD 20892, USA; 7 Neurobiology Neurodegeneration & Repair Laboratory (N-NRL), National Eye Institute, Bethesda, MD 20892, USAtiansen.li@nih.gov (T.L.); 8 National Eye Institute/NIH, Bethesda, MD 20892, USA; paulsieving@gmail.com

**Keywords:** optical coherence tomography (OCT), outer retina band, reflective intensity, mouse model, gene therapy, AMD patient

## Abstract

Optical coherence tomography (OCT) is widely used to probe retinal structure and function. This study investigated the outer retina band (ORB) pattern and reflective intensity for the region between bands 2 and 3 (Dip) in three mouse models of inherited retinal degeneration (Rs1KO, TTLL5KO, RPE65KO) and in human AMD patients from the A2A database. OCT images were manually graded, and reflectivity signals were used to calculate the Dip ratio. Qualitative analyses demonstrated the progressive merging band 2 and band 3 in all three mouse models, leading to a reduction in the Dip ratio compared to wildtype (WT) controls. Gene replacement therapy in Rs1KO mice reverted the ORB pattern to one resembling WT and increased the Dip ratio. The degree of anatomical rescue in these mice was highly correlated with level of transgenic RS1 expression and with the restoration of ERG b-wave amplitudes. While the inner retinal cavity was significantly enlarged in dark-adapted Rs1KO mice, the Dip ratio was not altered. A reduction of the Dip ratio was also detected in AMD patients compared with healthy controls and was also positively correlated with AMD severity on the AMD score. We propose that the ORB and Dip ratio can be used as non-invasive early biomarkers for retina health, which can be used to probe therapeutic gene expression and to evaluate the effectiveness of therapy.

## 1. Introduction

Optical coherence tomography (OCT) is widely used both in eye clinics and in research laboratories for the non-invasive investigation of retinal structure and abnormalities [1,2,3,4,5]. OCT imaging can be safely performed in young children and neonates [6,7] and can provide a high-resolution image that is very similar to histopathological specimens [8,9]. The OCT signal is produced by the interference strength of light reflected from the tissue with a reference light traveled at the same optical distance. Therefore, OCT signal can be used as a measure of different reflectivity for each tissue layer. Due to the laminar structure of the retina, OCT images are identified by bands of various reflectance intensity and those in the inner retinal structure have been well documented [10,11,12,13].

While all hyporeflective and hyperreflective bands in inner retina are identified and named [1], studies of outer retina bands (ORB) are commonly focused on four hyperreflective bands. ORB 1 and 4 represent the external limiting membrane (ELM) and retinal pigment epithelium (RPE), whereas the cellular origins for band 2 and band 3 are less certain [1,9,14,15,16,17,18,19]. It has been proposed that band 2 comes from either the inner segment ellipsoid zone [1] or inner segment/outer segment junction [20,21], and band 3 arises from the interdigitation of RPE microvilli with the photoreceptor outer segment tip [21]. On the other hand, the three intervening hyporeflective regions have attracted less attention [22]. For the simplicity of description in this study, we adopt the designations of band 2 and band 3 and refer to the hyporeflective region in between these two bands as “Dip”, as shown on the longitudinal reflectivity profile [23]. We introduce “Dip ratio” to quantitate the level of hyporeflectivity in this region.

There are many reports indicating that the ORB is altered in various diseased retinas, including inherited retinal diseases (IRD), diabetic retinopathy (DR), and age-related macular degeneration (AMD) [14,15,24,25], and that a relatively well-preserved ORB pattern is associated, accordingly, with better visual acuity [7,26,27,28]. A recent study has noticed an increase in reflectance in the Dip region, resulting in the merging of band 2 and band 3, in several mouse models of rod photoreceptor degeneration [29].

To further investigate whether the reflectance at the Dip region may serve as an early biomarker for retinal degeneration, we performed quantitative measurements of the Dip band in Rs1KO mice, a mouse model of human X-linked retinoschisis (XLRS). We evaluated the correlation between changes in ORB pattern and the levels of transgene expression after gene therapy to examine the Dip ratio as a biomarker for response to therapy. ORB patterns on OCT images from two other mouse IRD models (TTLL5KO and RPE65KO) were also analyzed. TTLL5 codes for tubulin tyrosine ligase like-5 (TTLL5), which glutamylates RPGR^ORF15^ in its Glu-Gly–rich repetitive region containing motifs in photoreceptors [30]. Lack of this enzyme leads to slow degeneration of rod photoreceptors. RPE65 codes for an isomerase in RPE cells that is critical for the visual cycle [31]. Mutation of this enzyme leads to the degeneration of both rod and cone photoreceptors. Three mouse IRD models (Rs1KO, RPE65KO, and TTLL5KO) used in this study represent photoreceptor degeneration at three different rates: fast, medium, and slow.

A similar analysis was extended to human subjects with retinal diseases. The A2A database compiled by the AREDS2 Ancillary SD-OCT Study [32,33], which includes AMD patients with their age-matched healthy controls, was used to determine the correlation of retinal disease with the Dip intensity in human subjects. AMD is characterized by drusen deposits, which usually localize in sub-RPE layers. To avoid potential artifacts induced by drusen through the compression of the retina tissue, we also examined Dip intensity in drusen-free regions in AMD patients. Our results indicate that Dip intensity is altered in AMD patients, both in drusen-overlaying and drusen-free retina regions. We propose that Dip intensity measured on OCT image can be used as a non-invasive early biomarker to evaluate the health status of the retina and efficacy of therapies.

## 2. Materials and Methods

### 2.1. Animals

This research was conducted in accordance with the ARVO Statement for the Use of Animals in Ophthalmic and Vision Research and was approved by the Animal Care and Use Committee of the National Eye Institute. All mice (Rs1KO [34], TTLL5KO [35], and RPE65KO [31]) were reared under 50 lux cyclic lighting (12 h:12 h) with food and water available ad libitum. Each mouse line was independently bred to a C57/B6 background, and wildtype (WT) littermates were used as respective controls. Number of mice used for each study is listed in Supplementary Table S1.

### 2.2. Optical Coherence Tomography Image Collection and Quantification

Mice were anesthetized by intraperitoneal injection of ketamine (100 mg/kg) and xylazine (10 mg/kg), and pupils were dilated with topical application of tropicamide and phenylephrine. OCT images were captured with a commercial Spectral-Domain OCT system (Envisu R2200, Bioptigen, Durham, NC, USA). Envisu system employs a wider bandwidth (160 nm) infrared light for OCT beam and provides an ultrahigh axial resolution (1.6 μm in tissue). The power of OCT beam on the cornea is less than 750 μW and is significantly below the ANSI standard for human ocular imaging [36]. Furthermore, the laser is always scanning, never stationary on one spot of the retina. It is not harmful to any ocular tissue. Radial volume scans, with optic nerve (ON) head centered, consisting of 4 B-scans (1000 A-scans per B-scan) were collected at 45 angular intervals and were each an average of five frames.

Two methods were used to quantitate ORB on mouse OCT images. The first approach was categorical analysis. For each B-scan, two sides of the ON head were scored separately for WT-like pattern (score 1), KO-like pattern (score 0), and mixed pattern (score 0.5) (Supplementary Figure S1). Therefore, radial volume scan consisting of 4 B-scans will have a total score of 8 for WT mouse eye, 0 for Rs1KO mouse eye, value between 0 and 8 for treated eye. The second approach was used to quantitate relative intensity of Dip band, as schematically illustrated in Figure S2. For each B-scan, 200-pixel (A-scan) region (350 to 630 um away from center of ON head) were used to measure reflectance intensity [22,37]. Within each region, OCT intensity was calculated after alignment of A-scan at ELM position. Outer nuclear layer (ONL) intensity was treated as background, and Dip ratio was calculated by peak intensity of band 2 divided by the intensity at Dip (valley between band 2 and band 3).

### 2.3. Intravitreal Injections, Immunofluorescent Assays, and RS1 Expression Level Evaluation

One eye each of eighty Rs1KO mice, aged between 21 and 49 postnatal days, was injected intravitreally with an AAV8-RS1 vector, as described previously [38]. The un-injected fellow eye served as control. One microliter of viral vector at a dose of 3 × 10^9^ to 1 × 10^10^ vector genomes was injected per eye. One week after OCT collection, mice were euthanized, eyes fixed in 4% paraformaldehyde for 2 h on ice and processed for cryosectioning [38]. For Rs1 protein staining, five sections (four vertical sections taken at evenly spaced intervals from the nasal margin of the retina to the optic nerve head and one section taken temporal to the optic nerve) were stained with a rabbit polyclonal RS1 antibody against the N terminus of retinoschisin (amino acid residues 24–37) [34,38]. Rs1 expression was evaluated by subjective grading of the intensity and extent of immunostaining using image editing software (Photoshop CS64) as described previously [38]. The average of these scores was used to determine the staining intensity for each eye [38].

### 2.4. Electroretinography (ERG)

Full-field ERG responses were recorded from dark-adapted Rs1KO mice two months post-AAV injection using Espion E2 system (Diagnosys LLC, Lowell, MA, USA) [38,39]. ERGs were recorded simultaneously from both eyes at bandwidth of 0.1 to 500 Hz. Dark-adapted ERGs were evoked by white flashes (−5.8 to 2.7 log scotopic cd-s/m^2^). A-wave amplitudes were measured from baseline to trough, and the b-waves were measured from the a-wave trough to the peak.

### 2.5. Human Control and Patients

OCT images from human subjects were selected from A2A database (AREDS2 Ancillary SD-OCT Study [33]. Table 1 lists subject information. Averaged horizontal and vertical B-scans (fovea-centered) were analyzed for each eye. For each averaged B-scan image, two regions of interest (ROI), each containing 200 A-scans (about 2.5 mm to 4.5 mm away from fovea) were selected. For each ROI, band 2 was manually segmented using ImageJ V1.53 (shown as red line in Supplementary Figure S3), and OCT intensity was averaged after aligned by band 2 position to generate intensity profile. For OCT images of AMD patients, retinal regions containing drusen were manually marked (shown as shaded areas in Supplementary Figure S3). OCT intensities for drusen-overlaying and drusen-free retinal regions were calculated separately.

### 2.6. Statistical Procedures

One-way ANOVA and Sidak’s multiple comparisons test were used to compare the ratio of band 2/Dip among the age groups of animal models. Wilcoxon matched pairs signed rank test was used to compare the ratio of band 2/Dip between treated and untreated eyes. Linear regression was used to model the relationship between ORB improvement scores and RS1 protein staining scores, ORB improvement scores and the ratio of ERG b/a wave amplitudes of treated over untreated eyes, and the correlation of these two-set data was evaluated using Pearson correlation coefficients. Two-way ANOVA with mixed-effects analysis and Dunnett’s multiple comparisons test were used to compare the ratio of band 2/dip among the humans’ conditions. Statistical analysis was performed using Prism (V9.5.1, GraphPad Software).

## 3. Results

### 3.1. RS1 Protein in the Retina and the Pattern of ORB in OCT Images

**Figure 1:**
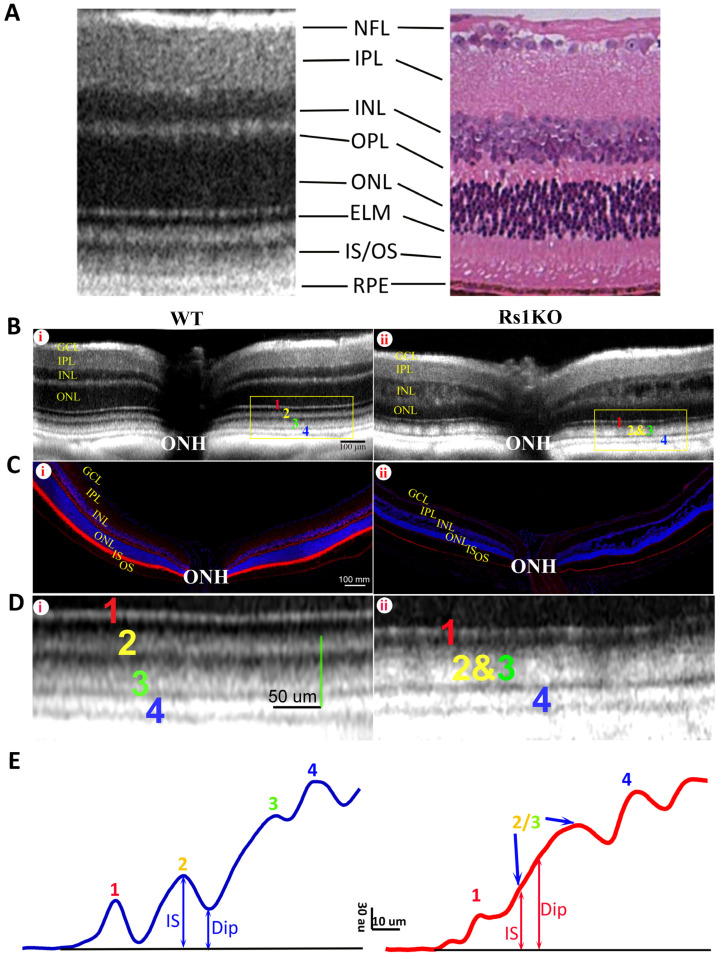
The OCT image and the histological immunofluorescent RS1 staining of 4 mo old WT and Rs1KO retinas. (**A**) Typical mouse retina OCT image (**left**) compared with a histologic section (**right**). Corresponding retinal layers are labelled: NFL, nerve fiber layer; IPL, inner plexiform layer; INL, inner nuclear layer; OPL, outer plexiform layer; ONL, outer nuclear layer; ELM, external limiting membrane; IS/OS, inner segment/outer segment; RPE, retinal pigment epithelium. (**B**) The OCT images of the WT (i) and Rs1KO mice (ii). Four hyperreflective outer retina bands (ORBs) are labeled with 1–4 (1: ELM; 2: IS/ep; 3: photoreceptor tip; 4: RPE), and they are clearly separated from each other in the WT retina with a distinct hyporeflective (Dip) region between bands 2 and 3. On the other hand, the Dip region between bands 2 and 3 has disappeared in the Rs1KO retina (Bii), leading to the merge of bands 2 and 3. ONH: optic nerve head. (**C**) The immunofluorescent RS staining of the WT retina (i) and Rs1KO retina (ii). Multiple layers of the WT retina are stained by the RS antibody, predominantly in the inner segment; the RS staining is completely absent in the Rs1KO retina. Faint fluorescent signal in the RPE layer is non-specific staining of the tissue. (**D**) Magnified OCT image of the region outlined by yellow boxes shown in B to better illustrate the ORB pattern in the WT (i) and Rs1KO (ii) retinas. (**E**) Average OCT intensity profiles for image shown in (**D**) illustrate the measurement of Dip and IS intensity; au: arbitrary unit of 8 bit gray-scale image. Dip ratio (peak intensity of band 2 divided by intensity of Dip) is high in the WT (**left**) but low in the Rs1KO (**right**) retina.

**Figure 2:**
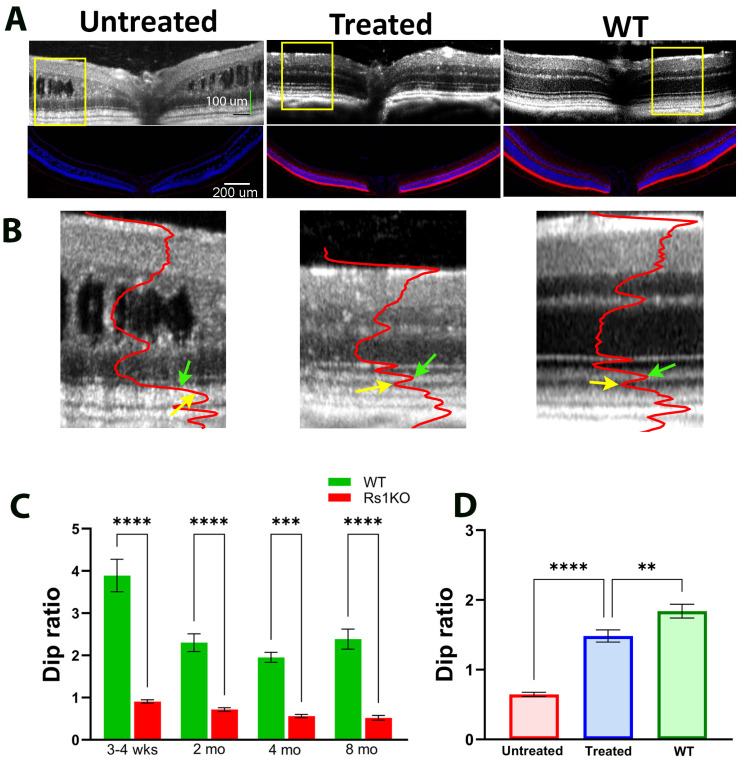
Improvement of OCT image pattern by gene therapy. (**A**) OCT images of an untreated, a treated Rs1KO mouse, and a WT mouse, with the corresponding immunofluorescent RS1 staining at the bottom panel. (**B**) Magnified view of OCT images derived from the yellow box region of 2A for untreated Rs1KO, treated Rs1KO, and WT mice. Superimposed red curve is the average intensity profile of the image. (Green arrows point to the peak of IS and yellow arrows point to the valley of dark band between band 2 and band 3). (**C**) Dip ratio measured at various time points for Rs1KO and WT mice. (**D**) Average Dip ratio for untreated Rs1KO, treated Rs1KO, and WT mice. (** *p* = 0.0032, *** *p* = 0.0008, and **** *p* < 0.0001).

Retinoschisin (RS) protein is dominantly expressed in the photoreceptor inner segment (IS) in mouse retina. Depleting RS protein results in a number of changes on retinal OCT images, including the development of cavities in the inner retina and an aberrant ORB pattern in the outer retina [9]. Figure 1 shows the OCT image (B) and RS immunoreactivity (C) of WT (Bi, Ci) and Rs1KO retinas (Bii, Cii). In WT mice, the OCT image of distal retina from ELM to RPE, the focus of this study, is characterized by four hyperreflective bands (Bi, numbers labelled) with three hyporeflective regions in between. A magnified view of the region marked by a yellow rectangle is shown in Figure 1D(i), with band 2 and band 3 clearly separated by a hyporeflective region (Dip). In contrast, in OCT images of Rs1KO retina, band 2 and band 3 are merged and the in-between hyporeflective region is missing (Bii). A magnified view of yellow-box region is shown in Figure 1D(ii). Immunostaining with RS1 antibody revealed expression of RS1 protein in multiple layers in the WT retina (Figure 1C(i)), with the highest expression in IS. RS1 expression is completely absent in the Rs1KO retina (Figure 1C(ii)). To quantitate the effect of RS1 loss on the ORB pattern, averaged intensity profiles were calculated from selected regions, and results are shown in Figure 1E left and Figure 1E right for the WT and Rs1KO retinas, respectively. For the WT retina, there is a clear valley for OCT intensity between band 2 and band 3, and the Dip ratio could be readily calculated based on minimal value in this region and peak intensity of band 2. For the Rs1KO retina, there is no clear valley for OCT intensity between band 2 and band 3. The Dip value had to be manually identified by the trend of OCT intensity increasing with the depth in retina, as illustrated in Figure 1E. This manually identified Dip point in the Rs1KO retina aligns well with the OCT intensity Dip in the WT retina (Supplementary Figure S4). The averaged Dip ratios for the WT and untreated Rs1KO retinas are shown in Figure 2C.

### 3.2. Improvement of the ORB Pattern in Rs1KO Mice after Gene Therapy

Rs1 Gene augmentation using AAV as a delivery vehicle reduced cavities in the inner retina and restored retinal function measured by ERG in Rs1KO mice [34,38,39]. In this study, we investigated if gene therapy could also affect the ORB pattern. Figure 2 shows examples of OCT images and immunofluorescence RS1 staining of WT and Rs1KO retinas after gene therapy. Figure 2A shows images of OCT (top) and matching immunostained RS1 (bottom) from the untreated (left) and treated (middle) retinas of an Rs1KO mouse, with a control WT retina shown on the right. While the RS1 protein is completely absent in the untreated Rs1KO retina, the treated retina shows an RS1 protein level and distribution pattern comparable to that of the WT retina. Figure 2B shows magnified OCT images of untreated (left), treated (middle), and WT (right) mouse eyes derived from the region marked by yellow boxes in Figure 2A, with superimposed averaged OCT intensity profiles. For untreated Rs1KO mice, the Dip ratio is already significantly lower than WT littermates at 3–4 weeks of age, the earliest time point examined (Figure 2C), and the Dip ratio gets progressively smaller with aging. In treated Rs1KO retinas, band 2 and band 3 are separated by a hyporeflective region, indicating that the Dip region has reemerged (Figure 2B). The averaged Dip ratio for these three groups of mice is summarized in Figure 2D.

### 3.3. Correlation of ORB Pattern with RS1 Expression Level and ERG Response

**Figure 3:**
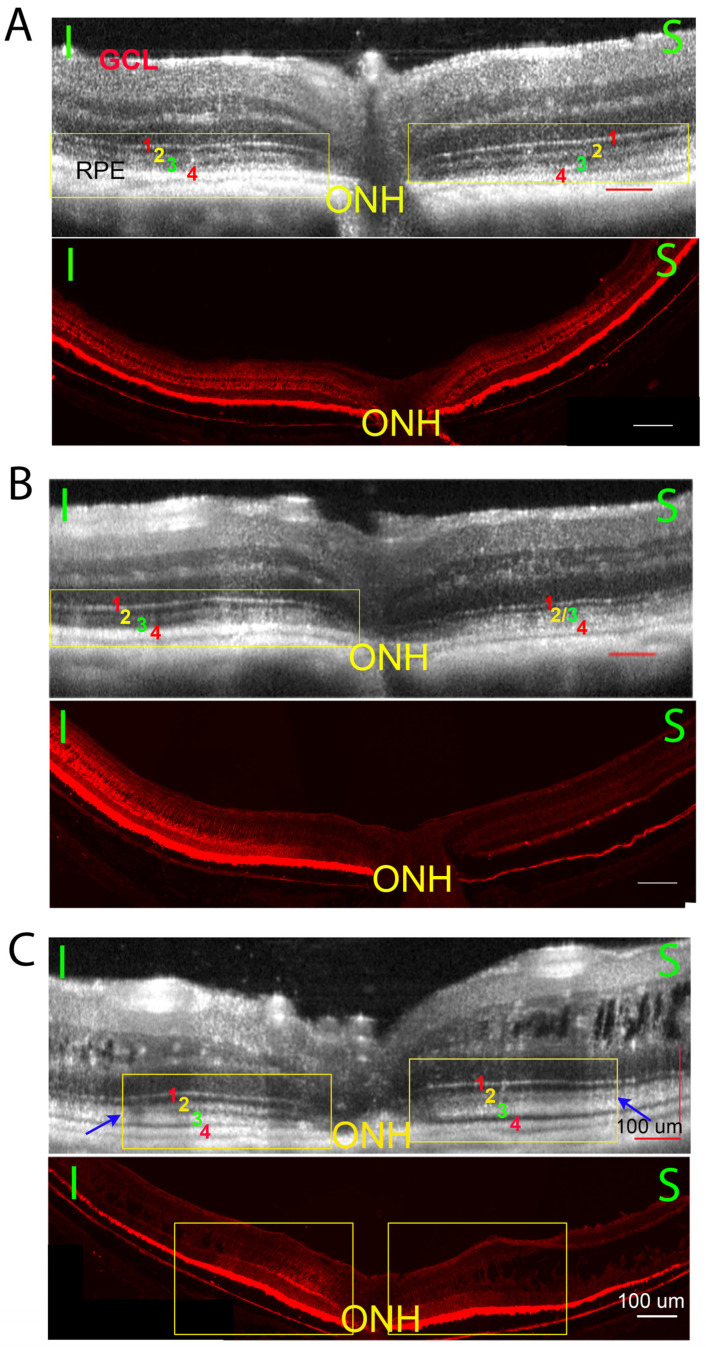
Three example pairs of OCT images (top images) and the transgene expression (bottom images) of histology sections from Rs1KO mouse eyes after gene therapy. While there are large variations in treatment efficiency, there is a high correlation between restoring the ORB pattern and the level of Rs1 expression. 3A, Retina on both sides of optic nerve head (ONH) showed ORB morphology improvement ((**A**) top, yellow rectangle) and extensive RS1 staining ((**A**), bottom); 3B, ORB morphology improvement ((**B**), top, yellow rectangle) and the extensive RS1 staining ((**B**), bottom) observed only on one side of ONH; 3C, ORB morphology is improved only at limited regions on both sides of the ONH ((**C**), top, yellow rectangle), and the extensive RS1 staining is also only observed on the corresponding area ((**C**), bottom, yellow rectangle). The OCT ORB improvement of the treated RSKO retinas is highly consistent with the extensive RS1 staining in the histology section. (I, inferior retina; S, superior retina).

Retinoschisin expression levels induced by intravitreal vector injection vary among the treated retinas [38]. Figure 3 shows three paired images that represent three different outcomes following gene delivery. For each panel, the top is the OCT image and bottom is the corresponding RS1 protein staining of the same eye. In panel A, a WT-like ORB pattern is observed on the OCT image on both sides of optic nerve head (ONH) (A, top image, yellow rectangle). A uniform RS1 staining is also detected across the entire retina (A, bottom image). In panel B, a WT-like ORB pattern is observed only on the left side of the retina (B, top image, yellow rectangle), matching stronger RS1 staining on the same side (B, bottom image). In panel C, a WT-like ORB pattern is observed flanking either side of ONH (C, top image, yellow rectangle), but fails to extend to the periphery. The blue arrows demarcate the margin where the WT-like ORB pattern transitions towards a KO-like pattern peripherally. The region of strong RS1 staining correlates closely with the WT-like ORB pattern (C, bottom image, yellow rectangle).

**Figure 4:**
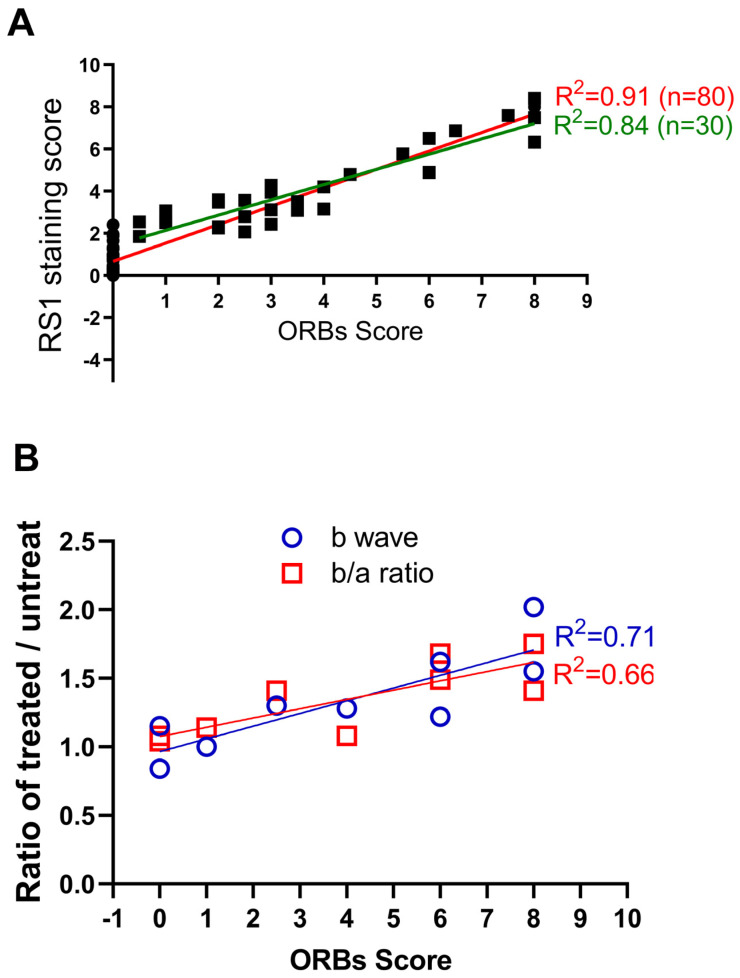
Correlation of ORB score with level of gene expression and functional recue. (**A**) The relation between the ORB improved score and the RS1 staining score. Lines are linear regression fit (red line: all data; green line, excluding near-zero points). (**B**) Relationship between the ORB improved score and the ratio of treated/untreated Rs1KO retinas in terms of b-wave amplitude, and b/a ratio.

To explore the relationship between the ORB pattern and RS1 protein expression level, we adopted a quantitative method to score ORB patterns from 0 (all KO-like) to 8 (all WT-like) (Supplementary Figure S1). RS1 protein levels were quantified using a previously published method [38]. Figure 4A shows the relationship between the level of RS1 expression and ORB score from eighty Rs1KO mice following gene supplementation. A linear fitting through all data points has an R square of 0.91 (red line; data excluding all near-zero points have an R square of 0.84, green line), indicating a high correlation between the RS1 expression level and the ORB score. In other words, improvements of the ORB pattern on the OCT image are highly dependent on the level of transgene expression.

We next explored whether an improvement of the ORB pattern correlates with retinal function. Previous studies indicate that gene therapy of Rs1KO mice mainly improves ERG b-wave amplitudes and the b/a wave ratio, but not a-wave amplitudes [38,39,40]. Therefore, we analyzed b-wave amplitude and the b/a ratio to assess functional improvement after gene therapy (Figure 4B). Outcomes are expressed as a ratio of values of treated over untreated eyes. We found a good correlation between the ORB score and ERG function for both b-wave amplitudes (R square is 0.71) and b/a ratio (R square is 0.66). Therefore, ORB scores derived from OCT images are predictive of retinal function after gene therapy.

### 3.4. Cavity Size in Rs1KO Mice

**Figure 5:**
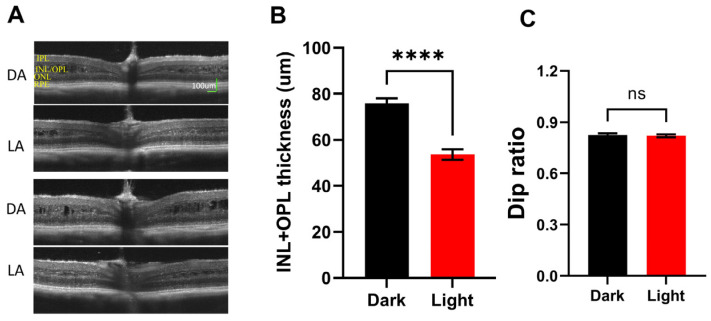
Effect of light adaptation on Rs1KO retina. (**A**) Two examples of OCT images captured after full-dark adaptation (DA) and the same eye after full-light adaptation (LA). Large cavity sizes on DA images should be noted. (**B**) Average inner retina thickness measured from dark-adapted OCT images and light-adapted OCT images from the same group of Rs1KO mice. (**C**) Dip ratio measured from the same set of dark-adapted and light-adapted OCT images. (**** *p* < 0.0001).

Retinoschisis is characterized by inner retina splitting (cavity formation). This pathological phenotype is recapitulated in rodent models created independently in several laboratories [34,41,42,43]. Therefore, cavity size in the retina is often used as one of the indices for disease progression [9]. We recently found that cavity size in Rs1KO mice is strongly influenced by lighting conditions. Figure 5A show two examples of OCT images captured under fully dark-adapted conditions (DA, top panel) and of the same mouse eye after fully adapting to room light (LA, ~500 Lux, bottom panel). In all twenty mice examined, dark-adapted eyes showed a much larger inner retina cavity than the same eye after light adaptation. The mechanisms mediating such light-induced cavity size changes are not clear. We used a combined thickness of inner plexiform to inner nuclear layer to quantitate cavity size in Rs1KO mice, and results are summarized in Figure 5B. While there is very significant differences in inner retina thickness (*p* < 0.0001), indicating large changes in cavity size by lighting conditions, the Dip ratio calculated from OCT images showed no statistical difference (*p* = 0.24, Figure 5C).

### 3.5. ORB Pattern in Other Mouse Models

**Figure 6:**
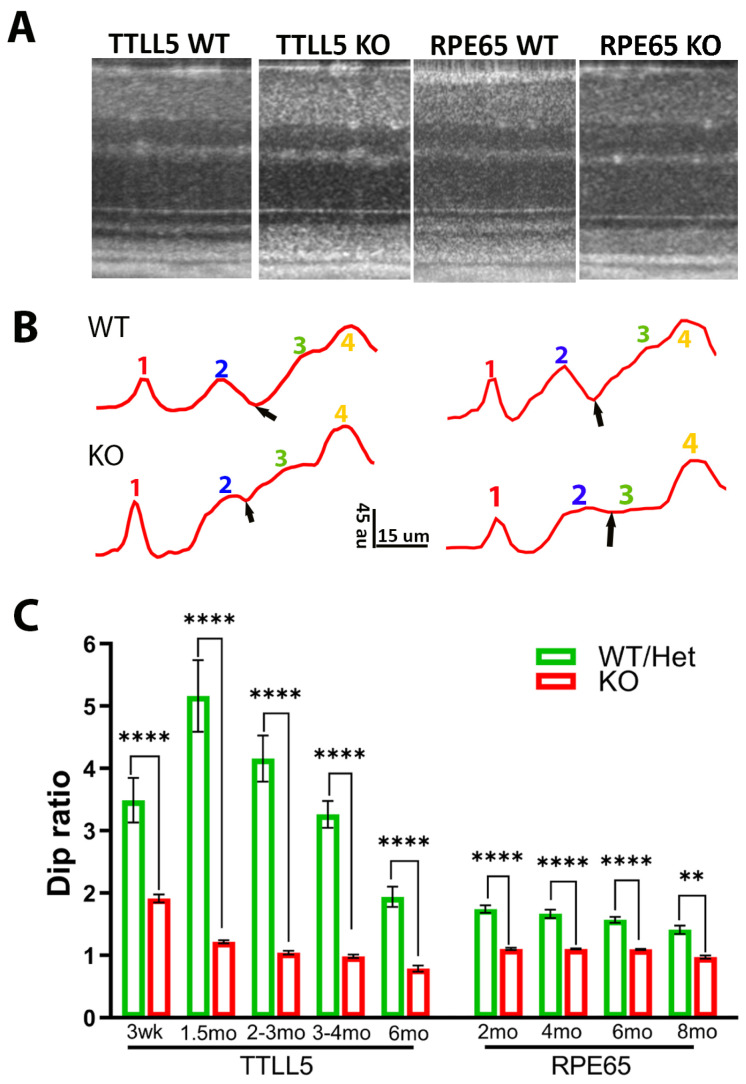
OCT image and Dip ratio in other mouse models. (**A**) Example of OCT image in WT, TTLL5 KO, and RPE65 KO mice. (**B**) Intensity profiles of outer retina region; small black arrows point to the position of Dip, au: arbitrary unit of 8 bit gray-scale image. (**C**) Dip ratio measured at various time points for TTLL5 KO and RPE65 KO mice and their respective WT littermates. (** *p* = 0.0034, **** *p* < 0.0001).

The ORB pattern of retinal OCT image was examined for two other mouse models of retinal degeneration: TTLL5KO and RPE65KO. Figure 6A illustrates examples of OCT images obtained from a TTLL5KO and a RPE65KO mouse, and their respective WT littermate controls. Figure 6B illustrates four ORBs intensity profiles. Figure 6C shows the Dip ratio derived from OCT images captured at various time points for mutant mice and their normal controls. For both mouse models, the difference between the KO and control groups’ Dip ratio was detected at the earliest time point examined (3–4 weeks for TTLL5 and 2 months for RPE65). The value was relatively stable after 2 months of age for both mouse models.

### 3.6. ORB Pattern in Human Patients

**Figure 7:**
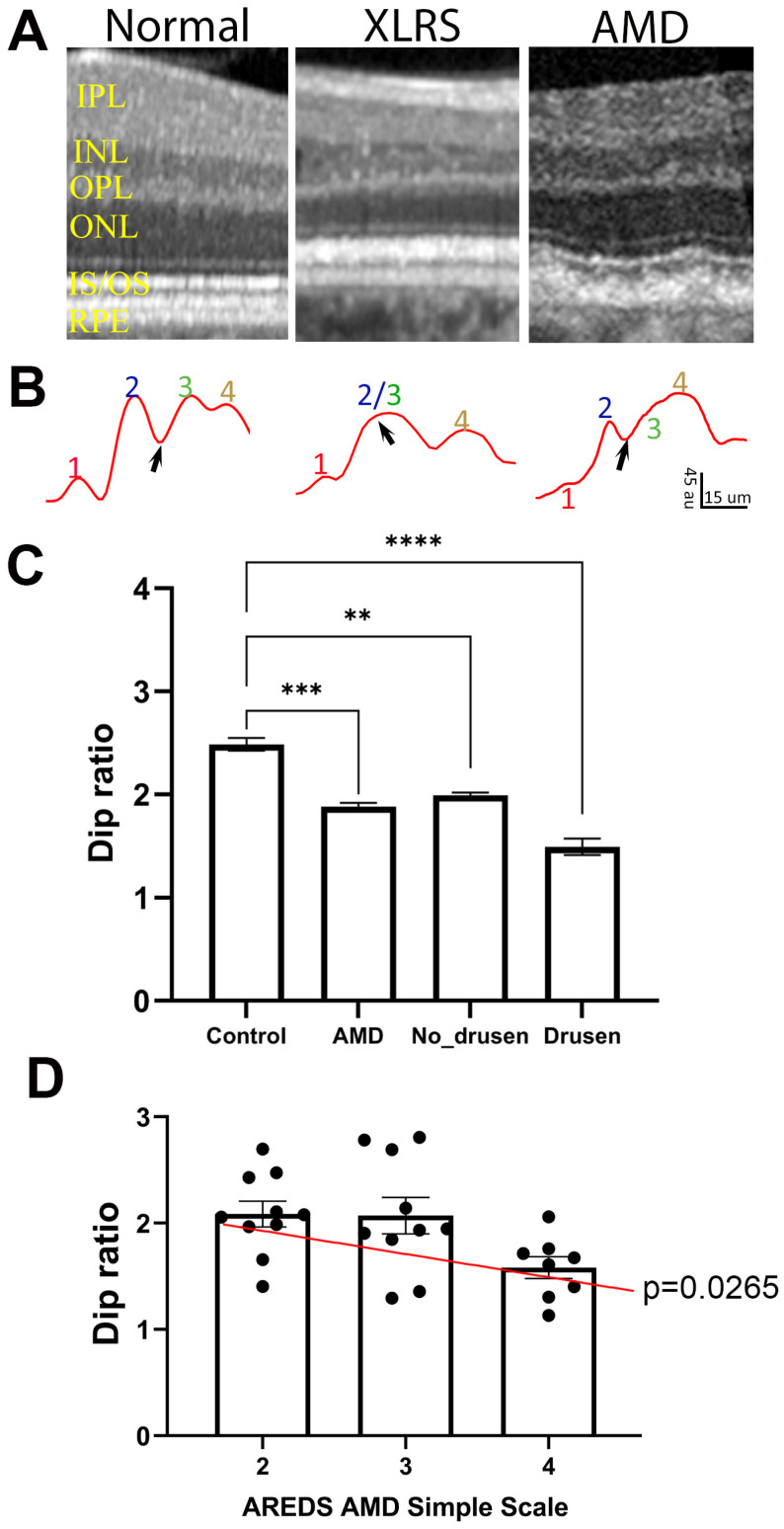
Dip ratio is low in human patients compared with healthy controls. (**A**) Example of OCT images in normal, XLRS, and AMD patients. (**B**) Intensity profiles of ORBs showing band 1 to 4, and Dip region (black arrows); au: arbitrary unit of 8 bit gray-scale image. (**C**) Dip ratio measure from OCT images of healthy (control) and AMD patients, and values measured from drusen-free (No-Drusen) and drusen-containing (Drusen) regions of AMD retinas. (**D**) Relationship of Dip ratio with AMD score for the patients. (** *p* = 0.0017, *** *p* = 0.0001, **** *p* < 0.0001). IPL, inner plexiform layer; INL, inner nuclear layer; OPL, outer plexiform layer; ONL, outer nuclear layer; IS/OS, inner segment/outer segment; RPE, retinal pigment epithelium.

We extended a similar analysis to OCT images captured from human subjects. Figure 7A shows examples of OCT images captured from a healthy control subject (Figure 7A, left), a patient with XLRS (Figure 7A, middle), and one with AMD (Figure 7A, right). Averaged OCT intensity profiles of OBRs are shown in Figure 7B. Similar to OCT images from WT mice, the healthy human subject also shows four hyperreflective bands separated by three hyporeflective bands, i.e., a clear presence of the Dip region between band 2 and band 3 (Figure 7B, left). However, for patients with retinal diseases, this Dip band is either narrow or absent on the OCT images (Figure 7B, middle and right).

To quantitate the effect of retinal disease on ORB patterns, we calculated the Dip ratio from OCT images obtained from the A2A database [33], which included AMD patients and age-matched healthy subjects. Table 1 lists the subject information included in this study. Only eyes with non-neovascular AMD were included, with the Age-Related Eye Disease Study (AREDS) AMD Simple Scale [32] of 2 to 4 (3.0 + 0.76). Similar to the mouse model of retinal diseases, the Dip ratio calculated from AMD patients showed significantly lower values than for the healthy controls (Figure 7C, *p* = 0.0001). As AMD is characterized by the presence of drusen in the sub-RPE space, which may deform the layer structure in the outer retina and affect Dip ratio calculation, we manually marked out drusen and no-drusen regions on the retina (Supplementary Figure S3) and calculated the Dip ratio for drusen-containing and no-drusen regions (Figure 7C). While drusen-containing regions showed lower Dip ratios than no-drusen regions, the Dip ratio of no-drusen regions of AMD patients was still significantly (*p* = 0.0017) lower than the Dip ratio obtained from healthy subjects. For AMD patients, there was also a positive correlation between the AREDS AMD Simple Scale and Dip ratio calculated from no-drusen regions (Figure 7D).

## 4. Discussion

In this study, we demonstrated stereotypical changes of ORB patterns in three mouse models of retinal degeneration with increases of Dip intensity in diseased animals compared to their littermate controls (Figure 1, Figure 2 and Figure 6). Our results, in combination with those reported by Joe et al. [29], support the notion that changes in ORBs are a common feature preceding rod photoreceptor degeneration in the retina. Using Rs1KO mice as a model, we also showed that ORB pattern and Dip intensity are highly correlated with expression levels produced by gene therapy and functional (ERG) outcomes (Figure 3 and Figure 4). Therefore, Dip intensity could be used as a non-invasive biomarker to probe gene expression in live eyes, which is particularly relevant for human study.

Our study also demonstrated that the changes in ORB pattern and Dip intensity are reversible in mouse models of retinal degeneration. Although Rs1KO mice showed low Dip ratios at the pretreatment stage, ORB patterns and Dip ratios returned to normal after successful expression of the therapeutic gene (Figure 2). This was also noted by Joe et al. for an X-linked RPGR mouse model [29]. Therefore, it is unlikely that a low Dip ratio reflects permanent structural damage to photoreceptors. Since rod degeneration mouse models and human patients exhibited similar changes on OCT images, we hypothesize that a low Dip ratio reflects stress conditions of the retina (such as pathology, malnutrition, oxidative stress, aging, etc.) and reversible structural changes. When such stress is released, such as after gene therapy, the Dip ratio returns to normal.

The cellular mechanisms for such ORB pattern changes are yet to be determined. The origin of band 2 has been proposed to arise from either the inner segment ellipsoid zone [1] or inner segment/outer segment junction [20,21], while band 3 seems to derive from the interdigitation of RPE microvilli into the photoreceptor outer segment tip [21]. The reason for the low reflectance of the Dip region between band 2 and band 3 in normal retinas is unclear. Even less is known about reduced Dip ratios in degenerative retinas, as there is no evidence to suggest increased interdigitation of RPE microvilli into photoreceptor outer segment regions in degenerative retinas, a proposed main source of reflectivity for band 3 [15]. On the other hand, the Dip region corresponds to the basal (proximal) outer segment with nascent discs. It is possible that these nascent discs are disorganized in diseased retinas and increase their reflectance. In addition, Zeng et al. [9] also noticed that mitochondria located in the inner segment ellipsoid zone are more disorganized in degenerative retinas than in normal controls. As mitochondria in the photoreceptor inner segment have light-focusing capacity [44], it is possible that disorganized mitochondria could alter the light path to reach the outer segment and, thus, change the band pattern on an OCT image.

On a few sample OCT images from human patients, we also noticed similar changes in the ORB pattern (Figure 7). The retrospective study with a human database showed a significant decline of Dip ratios in AMD patients. More interestingly, the Dip ratio is also significantly smaller for the drusen-free region of AMD retinas compared with healthy controls. Therefore, it is likely that the Dip value can serve as an index for retina stress before the retina shows pathological changes. This concept is also supported by the results obtained from TTLL5KO mice, which showed small Dip ratio at a young age, but photoreceptor degeneration happens very slowly and a significant reduction in the photoreceptor number is only detected in old animals [30].

As absolute intensity on OCT images can be influenced by many factors, such as the clarity of the optical media including lens and cornea, we used the Dip ratio of intensities at the Dip region and IS peak to quantitate changes. On the other hand, the OCT intensity of the IS peak could itself be affected by retinal diseases, especially under extensive degenerative conditions [1,45,46,47]. We measured IS peak intensity for the AMD patients and healthy controls used in this study (Supplementary Figure S5), and no statistical difference was noticed. Therefore, the difference of Dip ratios mainly reflect changes in the Dip region on OCT images.

As OCT images provide better spatial resolution than other functional examinations, such as ERG recordings, OCT biomarkers will be able to provide much more detailed information. ORB patterns and Dip ratios are also better biomarkers than other features noted on OCT images. For Rs1KO mice, the inner retina cavity is the hallmark of the disease, and is often used to monitor the progress of the disease [34,41,42,43]. However, cavities in the inner retina of Rs1KO mice often regress with age, whereas the retina continues to degenerate [9,39]. On the other hand, Dip ratios provide a better measure of the degenerative retina as their value continues to decrease with age for Rs1KO mice (Figure 2C). In addition, while cavity size can be significantly altered by lighting conditions, the Dip ratio is relatively constant (Figure 5).

Early detection of retinal disorders is critical and has a great impact on the prognosis and therapeutic intervention for the patients. Early diagnosis of inherited retinal diseases, such as X-linked retinoschisis, is often challenging with an average delay of 8 years after distinguishable symptoms become manifest [48]. Thus, it is often not possible to implement appropriate interventions at an early stage. Vision impairment caused by glaucoma and diabetic retinopathy, which are two leading causes of blindness [49], can be avoided if detected and treated at an early stage [50,51]. If the Dip ratio as an early biomarker is verified in more animal models and expanded to patients, it will help clinicians to diagnose retinal disorders and to evaluate the treatment.

## 5. Conclusions

Changes in the ORB pattern and Dip intensity on OCT images can be detected early in animal models of retinal degeneration before significant photoreceptor loss. The quantitative measurement of the Dip ratio can be used to evaluate the progress of retinal degeneration and effectiveness of the treatment. The improvement of Dip intensity can be used to predict the transgene level and functional rescue in an animal model. In addition, human patients also showed significant reduction in the Dip ratio, even from retinal regions lacking pathological markers. Our study supports the adoption of the ORB pattern and Dip intensity measured from OCT images as early biomarkers for retinal diseases and efficacy of treatment. Further studies are needed to confirm if this vital index for the transgene expression level and a valuable indicator for early detection of retinal disorders is also valid in other animal models and various forms of human retinal diseases.

## Figures and Tables

**Table 1:** Dataset for human subjects.

	Control	AMD
**Number**	23	24
**Eye**	45	29
**Male**	11	12
**Female**	12	12
**Age**	51–83 (66.7 ± 8.8)	61–86 (75.4 ± 6.0)
**AREDS AMD Simple Scale**	0	2–4 (3.0 ± 0.76)

## Data Availability

All relevant data are included in this manuscript (and its Supplementary Information files).

## Supplementary Materials

The following supporting information can be downloaded at https://www.mdpi.com/article/10.3390/bioengineering11050449/s1: Figure S1: Assess the improvement of the ORB in the treated retina after AAV8-hRS1 delivery; Figure S2: Schematic illustration of calculation of Dip ratio from mouse retina OCT image. Figure S3: Example of analyzed region of OCT from a human AMD patient; Figure S4: Normalized OCT intensity profile for WT and Rs1KO; Figure S5: IS intensity measured from control and AMD patients. Table S1: Number of mice used for each study.

## References

[1] Spaide R.F., A Curcio C. (2011). Anatomical correlates to the bands seen in the outer retina by optical coherence tomography: Literature review and model. Retina.

[2] Baghaie A., Yu Z., D’souza R.M. (2015). State-of-the-art in retinal optical coherence tomography image analysis. Quant. Imaging Med. Surg..

[3] Frohman E.M., Fujimoto J.G., Frohman T.C., A Calabresi P., Cutter G., Balcer L.J. (2008). Optical coherence tomography: A window into the mechanisms of multiple sclerosis. Nat. Clin. Prac. Neurol..

[4] Leung C.K. (2016). Optical Coherence Tomography Imaging for Glaucoma—Today and Tomorrow. Asia-Pacific J. Ophthalmol..

[5] Tsang S.H., Sharma T. (2018). Optical Coherence Tomography. Adv. Exp. Med. Biol..

[6] Rajjoub R.D., Trimboli-Heidler C., Waldman A.T., Avery R.A. (2015). Applications of optical coherence tomography in pediatric clinical neuroscience. Neuropediatrics.

[7] Ling K.P., Mangalesh S., Tran-Viet D., Gunther R., Toth C.A., Vajzovic L. (2020). Handheld Spectral Domain Optical Coherence Tomography Findings of X-Linked Retinoschisis in Early Childhood. Retina.

[8] Fischer M.D., Huber G., Beck S.C., Tanimoto N., Muehlfriedel R., Fahl E., Grimm C., Wenzel A., Remé C.E., van de Pavert S.A. (2009). Noninvasive, In Vivo assessment of mouse retinal structure using optical coherence tomography. PLoS ONE.

[9] Zeng Y., Petralia R.S., Vijayasarathy C., Wu Z., Hiriyanna S., Song H., Wang Y.-X., Sieving P.A., Bush R.A. (2016). Retinal Structure and Gene Therapy Outcome in Retinoschisin-Deficient Mice Assessed by Spectral-Domain Optical Coherence Tomography. Investig. Ophthalmol. Vis. Sci..

[10] Genead M.A., Fishman G.A., Anastasakis A. (2011). Spectral-domain OCT peripapillary retinal nerve fibre layer thickness measurements in patients with Stargardt disease. Br. J. Ophthalmol..

[11] Tatham, Andrew J., Felipe A. (2017). Detecting Structural Progression in Glaucoma with Optical Coherence Tomography. Ophthalmology.

[12] Hu Q.R., Huang L.Z., Chen X.L., Xia H.K., Li T.Q., Li X.X. (2017). X-Linked Retinoschisis in Juveniles: Follow-Up by Optical Coherence Tomography. BioMed Res. Int..

[13] Kwan C.C., Fawzi A.A. (2019). Imaging and Biomarkers in Diabetic Macular Edema and Diabetic Retinopathy. Curr. Diabetes Rep..

[14] Hood D.C., Zhang X., Ramachandran R., Talamini C.L., Raza A., Greenberg J.P., Sherman J., Tsang S.H., Birch D.G. (2011). The inner segment/outer segment border seen on optical coherence tomography is less intense in patients with diminished cone function. Investig. Ophthalmol. Vis. Sci..

[15] Park J.C., Collison F.T., Fishman G.A., Allikmets R., Zernant J., Liu M., McAnany J.J. (2015). Objective Analysis of Hyperreflective Outer Retinal Bands Imaged by Optical Coherence Tomography in Patients with Stargardt Disease. Investig. Ophthalmol. Vis. Sci..

[16] Spaide R.F. (2013). Outer retinal atrophy after regression of subretinal drusenoid deposits as a newly recognized form of late age-related macular degeneration. Retina.

[17] Ross D.H., Clark M.E., Godara P., Huisingh C., McGwin G., Owsley C., Litts K.M., Spaide R.F., Sloan K.R., Curcio C.A. (2015). RefMoB, a Reflectivity Feature Model-Based Automated Method for Measuring Four Outer Retinal Hyperreflective Bands in Optical Coherence Tomography. Investig. Ophthalmol. Vis. Sci..

[18] DeRamus M.L., Stacks D.A., Zhang Y., Huisingh C.E., McGwin G., Pittler S.J. (2017). GARP2 accelerates retinal degeneration in rod cGMP-gated cation channel beta-subunit knockout mice. Sci. Rep..

[19] Tanabu R., Sato K., Monai N., Yamauchi K., Gonome T., Xie Y., Takahashi S., Ishiguro S.-I., Nakazawa M. (2019). The findings of optical coherence tomography of retinal degeneration in relation to the morphological and electroretinographic features in RPE65−/− mice. PLoS ONE.

[20] Jonnal R.S., Kocaoglu O.P., Zawadzki R.J., Lee S.H., Werner J.S., Miller D.T. (2014). The cellular origins of the outer retinal bands in optical coherence tomography images. Investig. Ophthalmol. Vis. Sci..

[21] Yao X., Son T., Kim T.H., Le D. (2021). Interpretation of anatomic correlates of outer retinal bands in optical coherence tomography. Exp. Biol. Med..

[22] Gao S., Li Y., Bissig D., Cohen E.D., Podolsky R.H., Childers K.L., Vernon G., Chen S., Berkowitz B.A., Qian H. (2021). Functional regulation of an outer retina hyporeflective band on optical coherence tomography images. Sci. Rep..

[23] Huang Y., Cideciyan A.V., I Papastergiou G., Banin E., Semple-Rowland S.L., Milam A.H., Jacobson S.G. (1998). Relation of optical coherence tomography to microanatomy in normal and rd chickens. Investig. Ophthalmol. Vis. Sci..

[24] Acton J.H., Greenberg J.P., Greenstein V.C., Marsiglia M., Tabacaru Smith R., Tsang S.H. (2013). Evaluation of multimodal imaging in carriers of X-linked retinitis pigmentosa. Exp. Eye Res..

[25] Lima L.H., Sallum J.M.F., Spaide R.F. (2013). Outer retina analysis by optical coherence tomography in cone-rod dystrophy patients. Retina.

[26] Yang H.S., Lee J.B., Yoon Y.H., Lee J.Y. (2014). Correlation between spectral-domain oct findings and visual acuity in X-linked retinoschisis. Investig. Ophthalmol. Vis. Sci..

[27] Marco A., Bonini Filho M., Andre J., Witkin M.D. (2015). Outer Retinal Layers as Predictors of Vision Loss. Rev. Ophthalmol..

[28] Bennett L.D., Wang Y.Z., Klein M., Pennesi M.E., Jayasundera T., Birch D.G. (2016). Structure/Psychophysical Relationships in X-Linked Retinoschisis. Investig. Ophthalmol. Vis. Sci..

[29] Joe M.K., Li W., Hiriyanna S., Yu W., Shah S.A., Abu-Asab M., Qian H., Wu Z. (2019). A Common Outer Retinal Change in Retinal Degeneration by Optical Coherence Tomography Can Be Used to Assess Outcomes of Gene Therapy. Hum. Gene Ther..

[30] Sun X., Park J.H., Gumerson J., Wu Z., Swaroop A., Qian H., Roll-Mecak A., Li T. (2016). Loss of RPGR glutamylation underlies the pathogenic mechanism of retinal dystrophy caused by *TTLL5* mutations. Proc. Natl. Acad. Sci. USA.

[31] Redmond T.M., Yu S., Lee E., Bok D., Hamasaki D., Chen N., Goletz P., Ma J.X., Crouch R.K., Pfeifer K. (1998). Rpe65 is necessary for production of 11-cis-vitamin A in the retinal visual cycle. Nat. Genet..

[32] Ferris F.L., Davis M.D., Clemons T.E., Lee L.Y., Chew E.Y., Lindblad A.S., Milton R.C., Bressler S.B., Klein R.G., Age-related eye disease study research group (2005). a simplified severity scale for age-related macular degeneration: AREDS Report No. 18. Arch. Ophthalmol..

[33] Leuschen J.N., Schuman S.G., Winter K.P., McCall M.N., Wong W.T., Chew E.Y., Hwang T., Srivastava S., Sarin N., Clemons T. (2012). Spectral-Domain optical coherence tomography characteristics of intermediate age-related macular degeneration. Ophthalmology.

[34] Zeng Y., Takada Y., Kjellstrom S., Hiriyanna K., Tanikawa A., Wawrousek E., Smaoui N., Caruso R., Bush R.A., Sieving P.A. (2004). *RS-1* Gene Delivery to an Adult *Rs1h* Knockout Mouse Model Restores ERG b-Wave with Reversal of the Electronegative Waveform of X-Linked Retinoschisis. Investig. Ophthalmol. Vis. Sci..

[35] Lee G.S., He Y., Dougherty E.J., Jimenez-Movilla M., Avella M., Grullon S., Sharlin S., Guo C., Blackford J.A., Awasthi S. (2013). Disruption of Ttll5/stamp gene (tubulin tyrosine ligase-like protein 5/src-1 and tif2-associated modulatory protein gene) in male mice causes sperm malformation and infertility. J. Biol. Chem..

[36] (2022). American National Standard for Safe Use of Lasers.

[37] Li Y., Fariss R.N., Qian J.W., Cohen E.D., Qian H. (2016). Light-induced thickening of photoreceptor outer segment layer detected by ultra-high resolution oct imaging. Investig. Ophthalmol. Vis. Sci..

[38] Bush R.A., Zeng Y., Colosi P., Kjellstrom S., Hiriyanna S., Vijayasarathy C., Santos M., Li J., Wu Z., Sieving P.A. (2016). Preclinical Dose-Escalation Study of Intravitreal AAV-RS1 Gene Therapy in a Mouse Model of X-linked Retinoschisis: Dose-Dependent Expression and Improved Retinal Structure and Function. Hum. Gene Ther..

[39] Kjellstrom S., Bush R.A., Zeng Y., Takada Y., Sieving P.A. (2007). Retinoschisin gene therapy and natural history in The *rs1h*-KO mouse: Long-term rescue from retinal degeneration. Investig. Ophthalmol. Vis. Sci..

[40] Ou J., Vijayasarathy C., Ziccardi L., Chen S., Zeng Y., Marangoni D., Pope J.G., Bush R.A., Wu Z., Li W. (2015). Synaptic pathology and therapeutic repair in adult retinoschisis mouse by AAV-RS1 transfer. J. Clin. Investig..

[41] Chen D., Xu T., Tu M., Xu J., Zhou C., Cheng L., Yang R., Yang T., Zheng W., He X. (2017). Recapitulating X-Linked Juvenile Retinoschisis in Mouse Model by Knock-In Patient-Specific Novel Mutation. Front. Mol. Neurosci..

[42] Zeng Y., Qian H., Campos M.M., Li Y., Vijayasarathy C., Sieving P.A. (2022). Rs1h(−/y) exon 3-del rat model of X-linked retinoschisis with early onset and rapid phenotype is rescued by RS1 supplementation. Gene Ther..

[43] Weber B.H.F., Schrewe H., Molday L.L., Gehrig A., White K.L., Seeliger M.W., Jaissle G.B., Friedburg C., Tamm E., Molday R.S. (2002). Inactivation of the murine X-linked juvenile retinoschisis gene, Rs1h, suggests a role of retinoschisin in retinal cell layer organization and synaptic structure. Proc. Natl. Acad. Sci. USA.

[44] Ball J.M., Chen S., Li W. (2022). Mitochondria in cone photoreceptors act as microlenses to enhance photon delivery and confer directional sensitivity to light. Sci. Adv..

[45] Lee K.E., Heitkotter H., Carroll J. (2021). Challenges Associated with Ellipsoid Zone Intensity Measurements Using Optical Coherence Tomography. Transl. Vis. Sci. Technol..

[46] Cai C.X., Locke K.G., Ramachandran R., Birch D.G., Hood D.C. (2014). A comparison of progressive loss of the ellipsoid zone (EZ) band in autosomal dominant and X-linked retinitis pigmentosa. Investig. Ophthalmol. Vis. Sci..

[47] Staurenghi G., Sadda S., Chakravarthy U., Spaide R.F., International Nomenclature for Optical Coherence Tomography IN*OCT Panel (2014). Proposed lexicon for anatomic landmarks in normal posterior segment spectral-domain optical coherence tomography: The IN*OCT consensus. Ophthalmology.

[48] George N.D.L., Yates J.R., Moore A.T. (1996). Clinical features in affected males with X-linked retinoschisis. Arch. Ophthalmol..

[49] Flaxman S.R., Bourne R.R.A., Resnikoff S., Ackland P., Braithwaite T., Cicinelli M.V., Das A., Jonas J.B., Keeffe J., Kempen J.H. (2017). Global causes of blindness and distance vision impairment 1990–2020: A systematic review and meta-analysis. Lancet Glob. Health.

[50] Samagaio G., Estévez A., de Moura J., Novo J., Fernández M.I., Ortega M. (2018). Automatic macular edema identification and characterization using OCT images. Comput. Methods Programs Biomed.

[51] Rajagopalan N., Venkateswaran N., Josephraj A.N., Srithaladevi E. (2021). Diagnosis of retinal disorders from Optical Coherence Tomography images using CNN. PLoS ONE.

