# Supplementary material for: OCT Intensity of the Region between Outer Retina Band 2 and Band 3 as a Biomarker for Retinal Degeneration and Therapy
Source: Bioengineering (Basel). 2024 May 1;11(5):449. doi: 10.3390/bioengineering11050449 (PMC11118669; doi:10.3390/bioengineering11050449)
Supplement: Supplementary file 1 [file bioengineering-11-00449-s001.zip › bioengineering-2928181-supplementary.pdf]

Supplementary Material

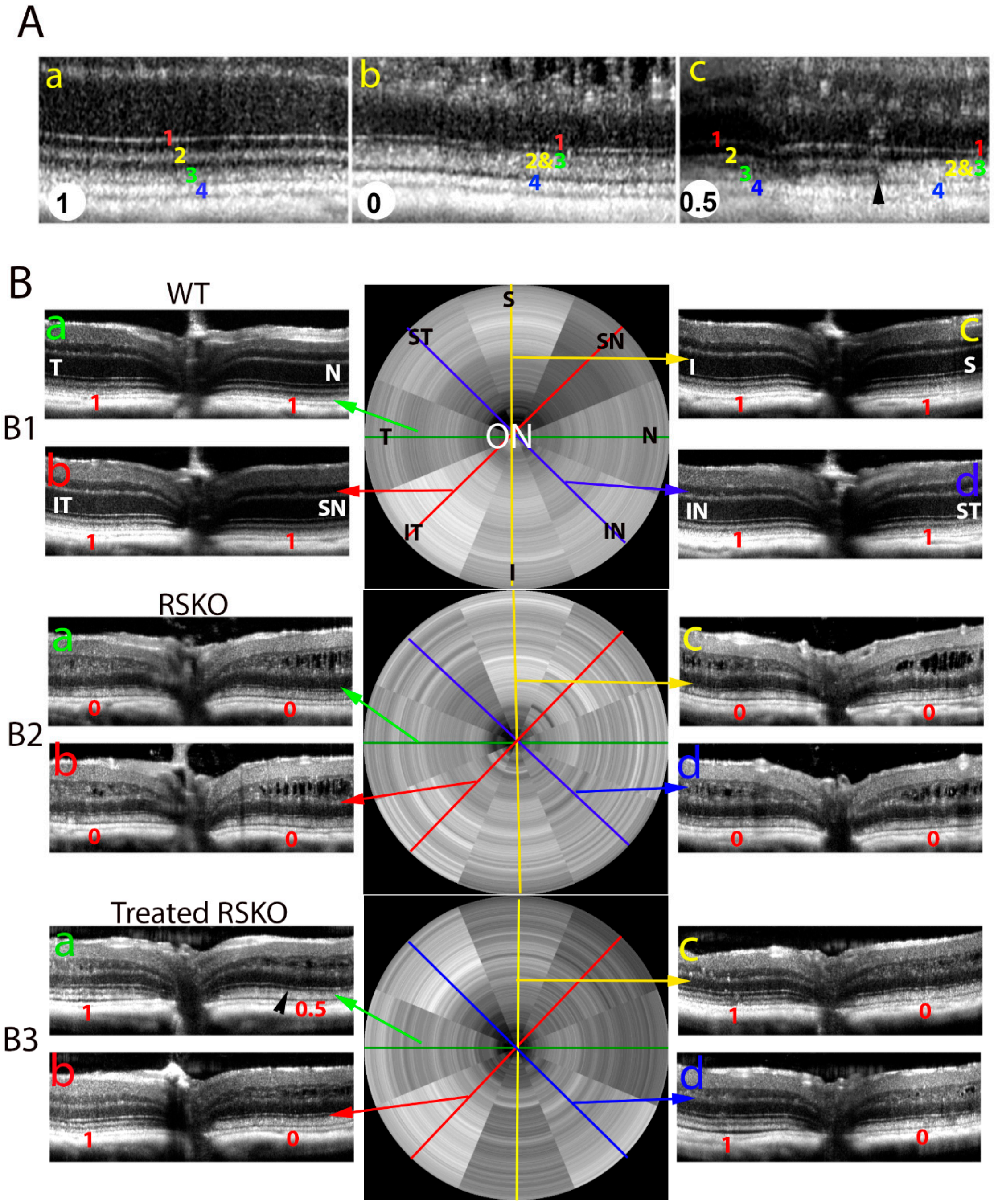

Supplement figure S1. How to quantitively assess the improvement of the ORB in the treated retina after AAV8-*hRS1* delivery. A, the ORB morphology of WT retina is given a score of 1 (left), the ORB morphology of *Rs1*KO retina is given a score of 0 (middle), the ORB morphology of treated *Rs1*KO that displays a partial improvement is given a score of 0.5 (right). Four B-scans of the radial scan obtained from WT, untreated *Rs1*KO, and treated *Rs1*KO retinas are listed in B, each B-scan is a cross-section of the retina centered by ON head, T to N cross-section (B1-a, B2-a, B3-a), IT to SN cross section (B1-b, B2-b, B3-b), I to S cross-section (B1- c, B2-c, B3-c), and IN to ST cross-section (B1-d, B2-d, B3-d), which are corresponding the region in middle images labeled with the same color lines(arrows point). B-B1, the ORB morphology of WT retain; B-B2, the ORB of untreated *Rs1*KO retina; B-B3, the ORB of treated *Rs1*KO retina. And the score obtained from each side of ON is labeled in each cross-section. ORB, outer retina band; I, inferior; S, superior; T, temporal; N, nasal; IT, inferior-temporal; ST, superior-nasal; SN, superior-nasal; IN, inferior-nasal.

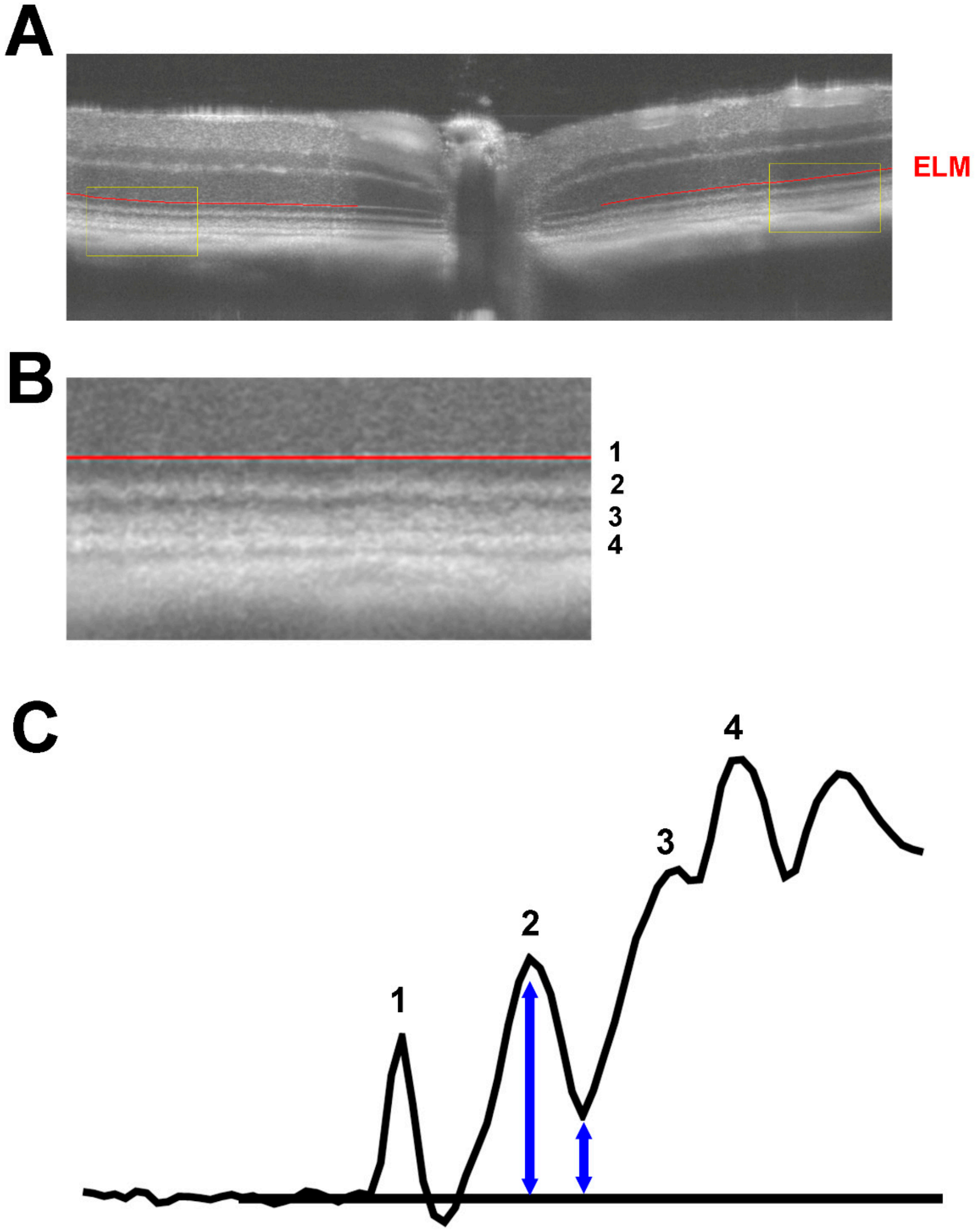

Supplement figure S2. Schematic illustration of calculation Dip ratio from mouse retina OCT image. A, Example of averaged Bscan OCT image with ELM layer identified by local maxima (redline). Yellow box on each side of OCT image marks analyzed region of interest (ROI). B, for each ROI, OCT Ascan is aligned at ELM line. Four outer retina band are labeled as 1-4. C, Average intensity profile is calculated ELM-aligned ROI image. Intensity at for band2 is identified by local maxima and intensity for Dip as local minima.

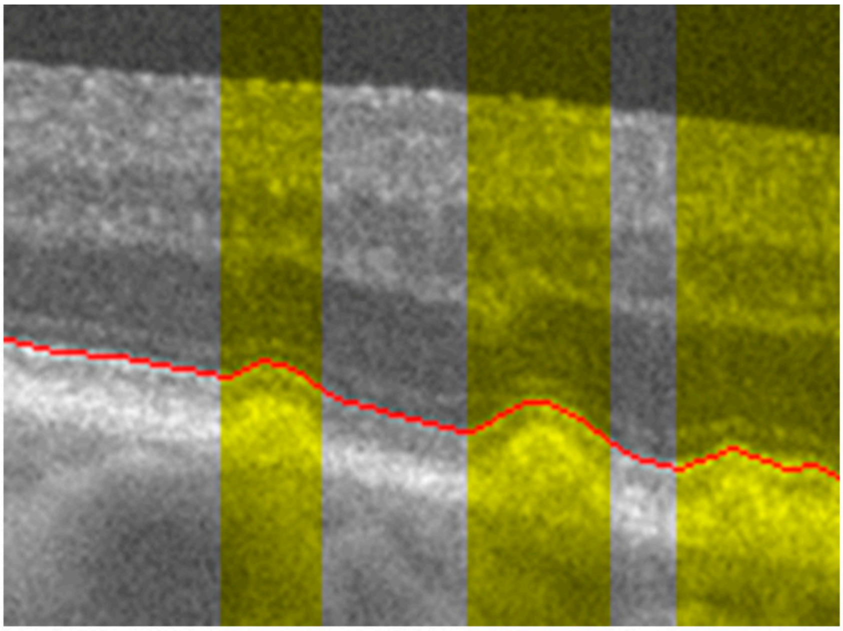

Supplement Figure S3: Example of analyzed region of OCT from a human AMD patient. Red line marks IS peak, and its position is used to align each Aline for averaging OCT intensity. Shaded region marks drusen-containing retina.

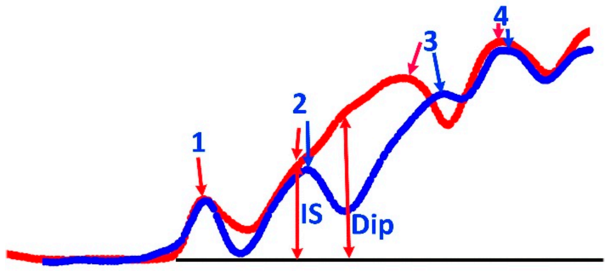

Supplement Figure S4: Normalized OCT intensity profile for WT (blue trace) and *Rs1*KO (red trace). Normalization was performed by align peak position of band 1 and band 4 (i.e. distance scale for *Rs1*KO trace is magnified). While Dip position in WT is identified as minimal intensity between band 2 and band 3, OCT intensity for *Rs1*KO also showed as transient point at the same location, which can easily be identified manually.

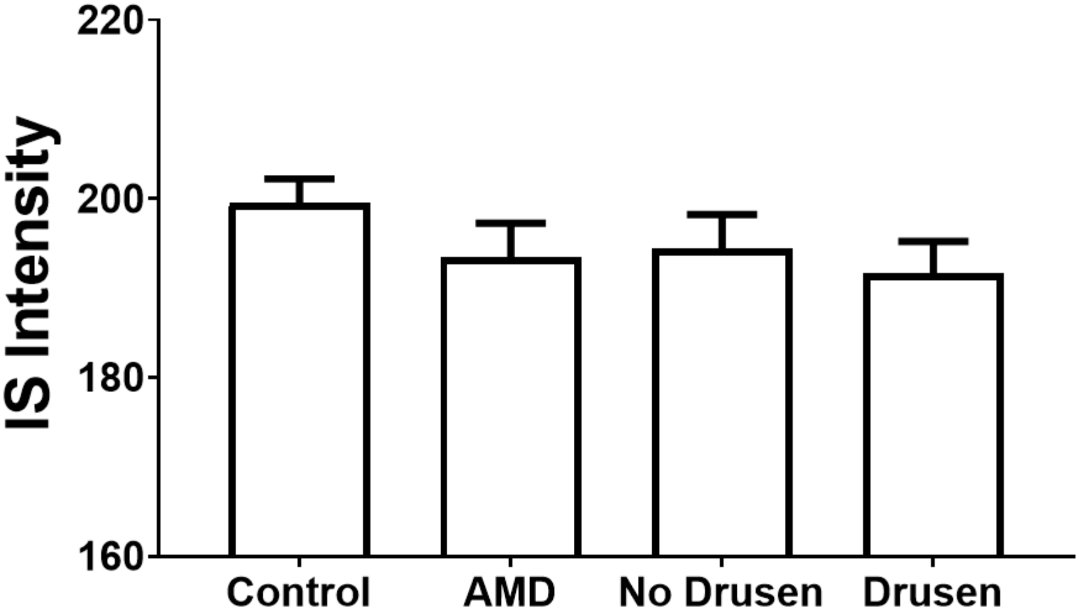

Supplement Figure S5: IS intensity measured from control and AMD patients. No significant difference was noticed.

Supplement Table S1: Number of mice used for each study.

Strains	Study	Number
Rs1KO	Gene therapy	80
	Dark & Light	20
	ERG	9
	Dip pattern	42
TTLL5 KO	Dip pattern	57
RPE65 KO	Dip pattern	53
